# The evolving therapeutic landscape of Graves’ disease in adults: present and future

**DOI:** 10.1530/ETJ-25-0078

**Published:** 2025-07-22

**Authors:** Marius N Stan, Chrysoula Dosiou

**Affiliations:** ^1^ Division of Endocrinology, Nutrition and Metabolism, Mayo Clinic, Rochester, Minnesota, USA; ^2^ Division of Endocrinology, Stanford University School of Medicine, Stanford, California, USA

**Keywords:** therapeutic, Graves’ disease, present, future

## Abstract

The therapeutic landscape of Graves’ hyperthyroidism has been rapidly evolving in the past few years. There has been a shift worldwide toward antithyroid drugs as the preferred first-line therapy with significant interest in thyroid function preservation, even if it requires more than 2 years of antithyroid drug treatment. This approach, long term antithyroid drug therapy, has gained traction as a therapeutic option after it has been shown to be safe and associated with significantly higher rates of remission than the traditional 18-month course of medical treatment. In parallel, we see, after 80 years of antithyroid drugs as the only medical therapy available for Graves’ disease, a strong interest in new drug development that follows more closely the pathophysiology of the disease. These approaches span the spectrum of targeting antigen presentation, B cell activation, TSHR antibody cycle and TSHR signaling. Separately, advances in wearable devices and artificial intelligence models present new opportunities for more timely diagnosis, monitoring, and treatment of patients with Graves’ disease. Finally, new therapies will pose novel challenges in the management of patients that will necessitate adjustments to our clinical practices and development of guidelines suited for these new therapeutic options.

## Introduction

### Epidemiology

Graves’ disease (GD) is an autoimmune disease that occurs in response to the development of autoantibodies against the TSH receptor (TSHR). It is the most common cause of hyperthyroidism, affecting between 0.5 and 1% of the population. It affects women much more frequently than men in a ratio of 8:1. It is frequently associated with clinical thyroid eye disease (TED) – about 25% of patients (1) – but uncommonly with other manifestations such as pretibial dermopathy and acropachy (10 and 2% of TED patients, respectively (2, 3)).

### Current therapies

Surgery is the oldest modality of treatment for GD and is still being used in 5–10% of cases today (e.g., for patients with large goiters, hyperthyroidism associated with concerning thyroid nodules) (4). A good approach to selecting preferred therapies for specific scenarios is outlined in the ATA hyperthyroidism guidelines (Table 5 of that publication) (4). Antithyroid drugs (ATDs) and radioactive iodine (RAI) treatment were advanced as therapies for GD in the early- to mid-1940s (5, 6). The use of these two options has varied significantly across regions of the globe, with preference for ATDs in Europe in contrast to preference for RAI as the first therapy in the United States (US) at that time (7). However, over the past decade, the utilization of these two therapies in the US has shifted significantly, as we will describe later. The choice of antithyroid agents has also shifted from PTU (favored before 2009) to methimazole and carbimazole after a report that indicated a much higher risk of liver toxicity with the former (8). Although PTU still remains a preferred choice during the first trimester of pregnancy and for those with minor allergic reactions to methimazole. It is also preferred for patients in active thyroid storm by ATA guidelines (4), while both PTU and MMI are considered effective for such by ETA (9) and JTA (10) guidelines. However, changing from one ATD to the other is contraindicated in patients that had any serious side effect from ATDs, given the potential cross-reactivity between these two drugs.

Most endocrinologists employ ATD therapy on a titration scheme aiming for restoring euthyroidism with the lowest possible dose of ATD, and thus minimizing the risk for adverse effects associated with higher doses of these drugs (11, 12). A less commonly employed ATD utilization is that of ‘block and replace’. This approach combines a higher dose of methimazole (that would induce hypothyroidism, as opposed to euthyroidism) with a dose of levothyroxine that restores normal thyroid levels. Its main utilization applies to patients with significant variability in thyroid hormone levels that fluctuate between hypothyroidism and hyperthyroidism. It is favored in several European countries but not endorsed in the US. While it is not known to increase the likelihood of a complete remission (13), it might be something that, in association with ongoing therapeutic research and in an altered version (TSHR blockade plus levothyroxine), will be potentially revisited (see section titled ‘Therapeutics of the future?’). Although safe and efficacious in controlling hyperthyroidism, ablative therapies do not address the underlying autoimmune pathophysiology of GD and cause lifelong hypothyroidism with associated need for levothyroxine therapy. Meanwhile, the ability of ATD therapy to address the autoimmune pathophysiology of GD remains debatable. Some studies have suggested a benefit from ATD in lowering thyrotropin receptor antibodies (TRAbs) (14), while others have refuted this action (15), in one case by identifying a similar remission between propranolol-treated patients and those treated with antithyroid drugs when studying mild disease (16). The main confounder remains the normalization of thyroid levels associated with ATDs and the possibility that it is the action that is followed by the beneficial impact on TRAb. From a practice perspective, if ATDs are indeed immunomodulatory, higher doses would be expected to improve remission rates. However, the vast majority of randomized-controlled trials (RCTs) have not supported this hypothesis (17, 18, 19, 20, 21).

## Current trends of therapy

### Change from RAI to ATD preference as first-line therapy

#### Physician survey data

**Figure 1:**
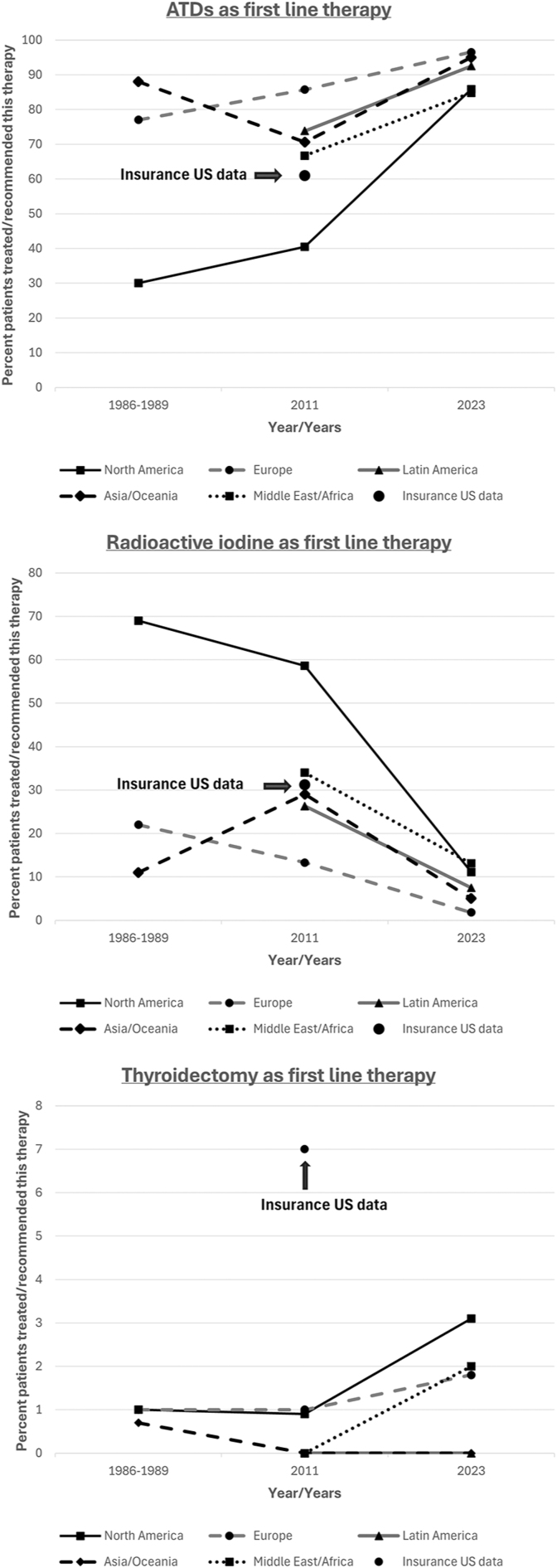
Changes in GD treatment choice over time. 1A – ATD therapy; 1B – radioactive iodine therapy; 1C – surgical therapy.

Clinical guidelines, such as the ATA 2016 hyperthyroidism guidelines, still advocate for selecting radioactive iodine, ATDs, or thyroidectomy as the first-line therapy in a shared decision model (4), while the NICE guidelines in the United Kingdom propose radioactive iodine as the first-line treatment in adults with GD, ‘unless ATDs are likely to achieve remission’ (22). Nevertheless, there have been significant changes in the practice patterns of endocrinologists treating hyperthyroidism in recent years (Fig. 1). Until the 1990s, RAI was the preferred treatment of choice in the US (23), in contrast to the rest of the world, where ATD therapy prevailed. Since the mid-2000s, however, the trend in the US has gradually changed, and practice patterns have shifted to the use of ATDs as the preferred first-line therapy, similar to the rest of the world ((24), Fig. 1). Worldwide surveys of members of different professional societies treating hyperthyroidism regarding therapy choices for an index patient with GD and five case variants highlighted these changes in practice. ATDs were the preferred primary mode of therapy worldwide in 54% of respondents in 2011 and in 92% in 2023. The differences were most dramatic in the US (41% in 2011 vs 86% in 2023), but the pattern was similar in other geographic areas (Europe 86% in 2011 vs 97% in 2023; Latin America 74% in 2011 vs 93% in 2023) (7, 25). The most important factor impacting therapy selection was the hope for remission, followed by hypothyroidism avoidance, avoidance of RAI, and avoidance of worsening TED (25). In the recent international survey, in aggregate geographic data, ATDs remained the preferred first-line treatment choice in variant case presentations, such as patients with TED (68%), planning pregnancy (73%), and in older age (81%). In a variant case presentation of a patient who relapsed 8 months after completing an 18-month course of ATD therapy following the achievement of an undetectable TRAb, ATDs were also still the preferred treatment (60%), with 40% of the physicians recommending definitive therapy (25).

#### Claims and medical record data and patient preference

Analysis of the actual claims and records of 4,661 patients (2005–2013) with GD indicated that 60% received ATDs as initial therapy, with 12% of those continuing ATDs beyond 2 years (26). Upon relapse after ATDs, 65% restarted ATDs, while 26% pursued RAI and 9% surgery. Overall, 26% of patients remained on ATDs for long term (first- and second-line therapy combined) (26). Similar initial choices were found in a Swedish study of 1,186 GD patients – 65% were initially treated with ATDs, but by the end of the 8 years follow-up, half of those had undergone ablative therapy (27). Consistently, when surveyed, both physician and patients preferred ATDs over RAI or surgery, (28). Interestingly, even though remission rates were the most important determinant of choice of therapy for patients and physicians alike, patients were willing to trade remission rates (accept a 38–44% decline) to receive ATDs over definitive therapy.

There are multiple reasons for the observed change in GD choice of therapy. We are now aware that long-term ATD (LTATD) therapy, defined as therapy for ≥2 years, has improved remission rates compared to standard therapy, with a favorable safety profile (29, 30). In addition, new studies showed prominent dissatisfaction in patients with hypothyroidism regarding their treatment (31, 32, 33). The shift away from RAI was also impacted by the data indicating increased risk of fetal/neonatal hyperthyroidism post-RAI, highest in pregnancies that occur 6–12 months after RAI (9%) (34). Other likely factors are concerns over potential malignancy risk associated with RAI (35) and greater awareness for increased risk of TED in relationship to RAI.

### Long-term ATD (LTATD) therapy

Traditional therapy with a 12–18 months course of ATDs carries a relapse rate around 50% (13, 36), with 9% of patients having relapse >10 years after the initial diagnosis (37). A systematic review from 2010, based on data from four prior RCTs comparing outcomes after treatment lasting from 6 months up to 42 months, concluded that ATD duration of therapy for >18 months did not improve relapse rates (13). Subsequent retrospective observational studies, however, supported the concept that longer duration of low-dose ATD treatment was associated with the greater chance of remission (38, 39, 40, 41). In a recent meta-analysis of six observational studies, with a mean therapy duration of 41–98 months, LTATD therapy (>24 months) was associated with a remission rate of 57%, with each additional year of treatment conferring an incremental 16% remission rate (CI 10–27%) (42). Azizi and colleagues later demonstrated that LTATD (additional 36–102 months after standard therapy) had a 4-year relapse rate after discontinuation of 15%, compared to 53% in the standard therapy group (29). It is noteworthy that the standard ATD group in this study had double the number of patients with large goiter compared with the LTATD group and a significant number of patients dropped out of the study, both aspects that could have significantly affected the chance of remission. LTATD therapy appears to be safe. The above RCT did not identify any ATD side effects after month 3 up to 120 months of treatment (43). Consistently, a review of 1,660 patients treated with ATDs for a mean of 5.8 years indicated only five adverse events after year 1 (30), while in a Danish multicenter study of LTATD users, after 24 months on 5 mg or less of methimazole, no reactions were recorded (44).

While existing ATA and ETA guidelines recommend consideration of LTATD as an alternative to definitive therapy in patients who relapse after a standard course of ATDs (4, 9), we do consider that this should be an option to discuss with all patients upfront, as suggested by other recent reviews (43, 45). Certainly, TRAb titers can be a useful guide in that ongoing conversation, acknowledging that TRAb seems to have multiple possible trajectories during the course of GD that clearly influence the patients’ chance of remission (46, 47). Overall, LTATD therapy is not a guaranteed remission success and certainly it applies only to a selected group of patients that tolerate ATDs well and are able to achieve euthyroidism with a low dose of these drugs.

### Prognostic scores for relapse

Given the high rate of relapse with standard ATD therapy, there have been persistent efforts over the years to predict such an outcome. The most validated (48, 49, 50) tool is that developed by Vos *et al.* that describes the risk for recurrence within 2 years of completion of 12 months of ATD therapy (51). The GREAT model uses clinical parameters alone (young age, large goiter, higher FT4 and higher TRAb titers) and separates three groups with risks between 16 and 68%. The same team also combined clinical + genetic variables (GREAT + model, using HLA and PTNP22) and identified four risk groups with relapse rates between 4 and 84%. The caveat of these results is the background use of ‘block and replace’ regimen, with discontinuation of therapy regardless of the TRAb titer after 12 months. These models are of value in patients that would not consider LTATD after standard ATD therapy. Two other models that try to predict the recurrence rate after ATD therapy are available but need additional validation (43, 52).

Further research into generating models with enhanced predictive value for recurrence, taking into account TRAb trajectories in addition to static values, or other newly identified risk factors such as IL-6 and TNFRSF9 levels (37), will be valuable in refining the role of ATDs, and particularly LTATD therapy in the therapeutic landscape of GD.

## Ideal goals of therapy

The initial goal of therapy for GD is to restore normal thyroid levels while, ideally, preserving normal thyroid parenchyma, given that the cause of thyrotoxicosis is related to autoimmunity, as opposed to an intrinsic thyroid pathology. As the disease infrequently enters into spontaneous remission (16), this target is reached through medical therapy currently. Achieving these two goals would eliminate the negative consequences of thyrotoxicosis and allow normal thyroid function to resume once the autoimmune process is controlled.

The second goal is achieving remission, i.e. the successful discontinuation of medical therapy with persistence of euthyroidism beyond 1 year of treatment (4) (ATA definition 2009 or 2016 guidelines). Ideally, this remission would be lifelong, but with current modalities, the rate is only around 50% (26).

The third goal is the elimination of TRAb. In addition to causing hyperthyroidism, these are thought to trigger the extrathyroidal manifestations of thyroid autoimmunity (TED, pretibial dermopathy – PTD, acropachy and neonatal thyrotoxicosis). Whether other autoantibodies are involved in the pathophysiology of these entities remains to be determined, and if proven as such, then their elimination should be part of the same goal.

The fourth goal is to achieve all the previous targets with therapies that are safe from the perspective of fertility, given that the patients affected by GD are relatively young, likely with interest in family planning.

## Therapeutics of the future?

**Figure 2:**
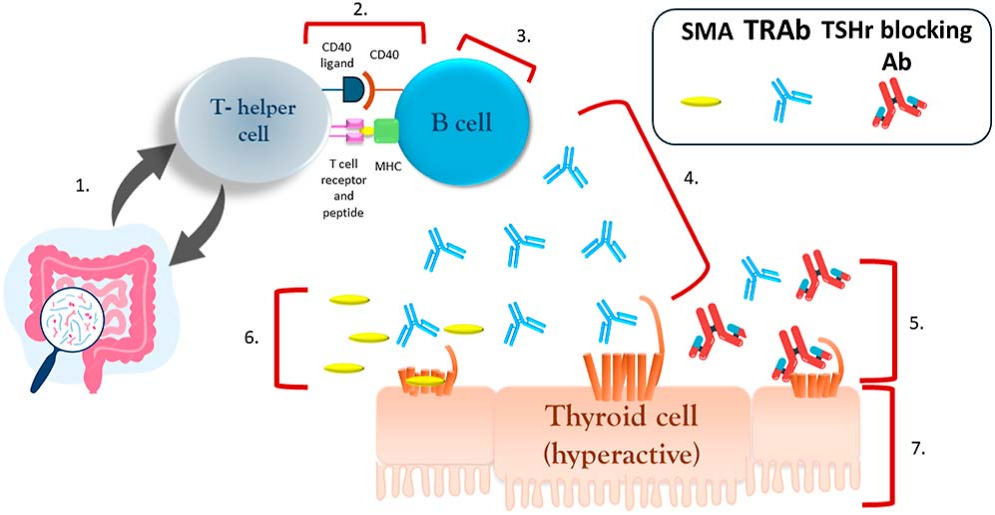
GD therapeutic targets under consideration. 1. Microbiome targeting (e.g. antibiotics); 2. B-cell activation targeting (e.g. iscalimab); 3. B-cell targeting (e.g. rituximab); 4. TRAb targeting (e.g. FcRn inhibition); 5. extracellular TSHR targeting (e.g. K 1-70); 6. transmembrane TSHR targeting (e.g. TSHR small-molecule antagonists); 7. non-surgical thyroid cell targeting (e.g. thermal ablation with RFA, HIFU). TSHR blocking Ab, TSH receptor blocking antibody (red/blue); SMA, small-molecule antagonist (yellow); TRAb, thyrotropin receptor antibody (blue); RFA, radiofrequency ablation; HIFU, high-intensity focused ultrasound.

In this section (and summarized in Fig. 2), we aim to describe several therapeutic approaches that are at various stages of exploration. Conversely, here, we do not describe the concepts that are being discussed in the field but are not yet substantiated by published data, neither do we discuss the therapies targeting TED specifically, without a known or expected impact on thyroid hormone levels. We hope these approaches will enrich our management armamentarium in the coming years, particularly as some of them have started to be investigated in humans (Table 1).

**Table 1:** Novel therapeutics with potential applicability in GD – clinical trial status.

Agent	Mechanism of action	Clinical trial status	Trial number
K1-70	TSHR blockade	Phase 1 in GD completed	NCT02904330
FcRn blockers			
Batoclimab	FcRn blockade	Open label study in GD, preliminary data presented ATA 2024	NCT05907668
		Phase 2a in TED completed	NCT03922321
		Phase 2b in TED completed	NCT03938545
		In phase 3 in TED	NCT05524571
IMVT-1402	FcRn blockade	In phase 2b in GD	NCT06727604
Efgartigimod*	FcRn blockade	In phase 3 in TED	NCT06307613
Iscalimab	CD40 blockade	Phase 2 in GD completed	NCT02713256
Tolerogenic therapy (ATX-GD-59)	Induction of tolerance	Phase 1 in GD completed	NCT02973802
		Phase 2 in GD suspended	NCT06240455

* FDA-approved for myasthenia gravis.

### TSHR blockade

The TSHR is the main target for the autoimmune response. Therefore, blocking this target would have a meaningful impact on reversing the manifestations of GD. The first agent utilized for this purpose was derived from the autoantibodies obtained from a patient with GD by the team of B R Smith (53). After encouraging preclinical data (54, 55), this monoclonal antibody, currently known as K1-70, has been utilized in two clinical projects. The first was an interesting case report where the antibody was administered to an individual with GD and TED in the setting of metastatic follicular thyroid carcinoma with lung metastases progressing (consistent with the elevated TRAb stimulation) despite high avidity for radioactive iodine (56). The use of K1-70 was followed by a positive response regarding both lung lesions and TED. Soon thereafter, the first open label clinical trial with this agent was reported (57). K1-70 was well-tolerated and controlled symptoms of hyperthyroidism with fairly rapid induction of biochemical hypothyroidism. Furthermore, most patients with TED also had improvement in their eye manifestations. Certainly, an RCT is desired to understand better the potential of this drug, but from these preliminary data, it appears that simultaneous replacement of thyroid hormone with levothyroxine is likely to be necessary to counteract rapid development of hypothyroidism.

The other modalities under exploration for blocking the TSHR are small molecule antagonists (SMAs). These compounds target the allosteric pocket located in the transmembrane region of the TSHR and could theoretically be administered orally. They appear to inhibit the conformational changes required for TSHR activation, even though they do not block the binding of stimulating TRAb. A few candidates are being explored (e.g. S37a (58), ANTAG3 (59), VA-K-14 (60), and SYD5115 (61)). The available data about their efficacy is relating to cell culture studies, where they are able to inhibit cyclic AMP production induced by M22, the commercially available TSHR-stimulating monoclonal antibody. The challenge with such agents relates to the similarity between the TSHR and LH/FSH receptors, necessitating high specificity for the TSHR to avoid disruption of the sex hormone regulatory loop. Potential off-target effects on other yet unknown GPCRs with such agents are a possible risk, likely not shared with TSHR-blocking antibodies as evidenced by the limited data with K1-70 (55).

### FcRn blockade

Elimination of the pathogenic TSH receptor autoantibodies is also being explored as a therapeutic approach through blocking FcRn, a recycling mechanism for immunoglobulins that prevents their degradation and extends their half-life. The benefit of such an approach would go beyond the control of hyperthyroidism and prove prophylactic or therapeutic for other TRAb-dependent autoimmune conditions. At least five FcRn inhibitors are in development. Batoclimab (IMVT-1401), a monoclonal antibody against FcRn, is the one most tested in thyroid autoimmune pathology. It showed promising results in TED patients in a phase 2b, multicenter placebo-controlled trial that unfortunately had to be stopped early due to the unexpected development of hypercholesterolemia (62). Besides the trend for reduction in proptosis, there was an early (2 weeks) dose-related 30–50% reduction in IgG with a simultaneous reduction in FT4 and FT3 levels as early as 1–2 weeks after treatment. Based on these data, a proof of concept, open label study of batoclimab in Graves’ patients (NCT05907668) reported ATD discontinuation in 56% of patients at 12 weeks (data presented at the ATA Annual Meeting, Chicago, 2024). The main adverse events seen with batoclimab related to the fact that it also interferes with albumin binding, causing a reduction in albumin with subsequent edema and hypercholesterolemia. IMVT-1402, a modified version of batoclimab that does not interfere with FcRn binding of albumin, is currently in phase 2b clinical trial (actively enrolling) in GD patients (NCT06727604). Another similar agent explored in thyroid autoimmunity is efgartigimod. This is an engineered IgG1 Fc fragment, which has no interaction with the albumin-binding site and has been FDA-approved for the treatment of myasthenia gravis. It is currently in phase 3 TED-focused clinical trials that are actively recruiting (NCT06307613). Other FcRn inhibitors that have not yet been evaluated in GD or TED include rozanolixizumab, nipocalimab, and orilanolimab.

### CD 40 blockade

The CD 40–CD 40 ligand interaction is a necessary stimulus for the activation of T helper cells. Inhibiting this interaction was explored as a way of modulating the autoimmune response (63). The agent tested for this was iscalimab, a monoclonal antibody that was also investigated for liver and kidney transplantation, Sjogren’s disease, SLE and other autoimmune pathologies. The open label series for patients with GD reported mixed responses with only roughly half of the patients achieving improvement/normalization of thyroid values. A low TRAb (<10 IU/L) predicted a good response while subsequent work suggested the possibility that genetic polymorphism of CD 40 might also predict response to this therapy (64).

### Tolerogenic therapy

The primary inciting event in GD pathogenesis is the breakdown of tolerance against the TSHR. Restoration of immune tolerance towards the TSHR has been recently explored. Applying the allergology principles of inducing tolerance to autoantigens has led to the creation of ‘apitopes’: synthetic peptides that mimic epitopes but are independent of antigen processing; this makes them less likely to stimulate the immune system. ATX-GD-59 is a TSHR apitope, a mixture of two peptides from the TSHR extracellular domain that binds with high affinity to HLA-DR molecules. It has been investigated in a phase 1 study of 12 patients with untreated GD (65). Subjects of specific MHC allelotypes were treated with ten intradermal injections of the apitope over the span of 18 weeks. Seven of the ten subjects that finished the study (out of 12) had improvement in thyroid function, with 50% normalizing FT3 concentrations. Treatment was reportedly well-tolerated. Further studies are needed to understand the full potential of this therapy, which would be an attractive option given the lack of systemic immunosuppression and targeted nature of the immune intervention. However, a planned phase 2 clinical trial with this agent (NCT06240455) has been suspended.

### Other immunomodulatory agents

A variety of immunomodulatory agents have been utilized in GD over the years. In a retrospective study of 162 patients treated with ATDs, addition of IV steroids for TED (*n* = 43) significantly decreased the risk of hyperthyroidism relapse in patients <40 years of age (66). Addition of methotrexate to ATD therapy in a RCT of 144 patients with GD resulted in higher rates of discontinuation of ATDs at 15 and 18 months compared with ATDs alone and greater reductions in TRAb levels from baseline starting at 9 months of treatment (67). In another randomized trial of 270 patients, addition of azathioprine to carbimazole was associated with higher remission rates and longer time to relapse compared with carbimazole monotherapy (68). There are also limited data in small pediatric and adult cohorts suggesting that rituximab, a B-cell depleting agent, improves remission rates when combined with methimazole (69, 70). While interesting, none of these studies have generated significant interest in changing the clinical paradigm of GD therapy. Considering the efficacy of MMI, its oral route, low price and its limited and well-known side effect profile, these non-standard therapies appear unable to claim superiority over the ATD-based therapeutic approach.

### Microbiome manipulation

The gut microbiome has been implicated in the pathogenesis of autoimmune disease through many mechanisms, including influencing differentiation of immature T cells residing in the gut and modulating intestinal mucosal permeability in ways that can trigger inflammation (71). The recent INDIGO study confirmed prior findings seen in an animal GD model of an increased ratio of *Firmicutes* to *Bacteroidetes* species in the fecal microbiome of patients with GD compared with healthy controls, while the presence of *Clostridiales* bacteria at diagnosis correlated with the persistence of TRAbs more than 200 days after commencing ATDs (72). Manipulation of the gut microbiome has been shown to successfully influence the incidence and severity of GD in a mouse model (73). Unfortunately, the animal models have not been consistent in their phenotype of hyperthyroidism and future studies are needed in humans to assess whether this could be a viable therapeutic or preventative intervention.

### Non-RAI thyroid ablation

Several groups have tried to eliminate the thyroid parenchyma through ablative therapies that are non-radioactive. High-intensity focused ultrasound (HIFU) is concentrating the energy of ultrasound waves at a distant target, inducing vibration and heat, which leads to tissue necrosis. It has been explored as a treatment for GD in Hong Kong (74). After a 2-year follow-up, the reported relapse rate in these partially ablated glands (avoiding the tracheo-esophageal groove and peri-carotid area) was around 40%, while no patients developed hypothyroidism. No comparative data have been reported from other centers, and longer-term follow-up is not available since the publication from 5 years ago. Given the complex set-up required and the high relapse rate, it is unlikely that this approach will prove useful at a large scale. The same group recently reported results of GD ablation using RFA (75). Their 2-year relapse rate was slightly above 40% and patients with smaller glands were more likely to have a longer remission. Microwave ablation is the third approach used by several groups to eliminate hyperthyroidism. Overall, the data on these ablative techniques are very sparse, no long-term follow-up is available, and since the ablations are subtotal, they are likely to be associated with suboptimal outcomes, similar to subtotal thyroidectomy as a treatment of option (76). In addition, there are some case reports describing GD development after RFA of thyroid nodules (77), raising concerns about these ablative modalities as a possible stimulator of autoimmunity.

## How will the overall management of GD change?

### Disease monitoring and therapy – role of technology

The management of hyperthyroidism during active therapy (with ATD) or of hypothyroidism post-RAI ablation or surgery is based on fixed time intervals at which thyroid levels are checked and the dose of ATD or levothyroxine is adjusted. However, the thyroid levels do not always respond in such a predictable fashion and the timelines we select for repeat testing are often arbitrary (ATA guidelines (4) suggest intervals between 2 weeks and 6 months depending on the duration of therapy). On the other hand, we have biological parameters that are a fairly accurate reflection of the metabolic status, and consequently of the thyroid status. Heart rate and temperature are easy to monitor, and both are readily available on many wearable devices. They have been occasionally used to identify GD (78), and under the assumption that they can identify changes of the metabolic rate, they can also indicate the need and timing for thyroid reevaluation. Such an approach has been tested prospectively by a group in South Korea (79) and could be a very promising tool to allow the individualization of therapy with significant improvement in the percent of time patients are euthyroid during active therapy, and earlier detection of recurrence, if that occurs. The use of technology for disease management does not stop at the utilization of biomarkers. There are efforts to better select the dose of methimazole for an individual based on mathematical modeling of available large datasets, rather than clinical experience (NCT06327828). Hopefully, promising results will be available in the near future. For more immediate clinical use are the predictive instruments of GREAT and GREAT+ (51) discussed earlier, which can indicate the probability of remission after 18 months of ATD therapy, a useful tool for patients in therapy selection.

### Challenges of new medical therapies

New medical therapeutics for GD will introduce new challenges in management. Some are common challenges for all new agents: i) proving long-term efficacy and establishing whether they are disease-modifying or just temporarily controlling the disease, ii) identifying their adverse event profile and its management, iii) assessing their safety around issues related to reproduction, and iv) controlling cost, which is likely to be much higher than ATDs. Therefore, justification of their use must be correlated with better long-term remission rates, decrease in associated comorbidities (TED/PTD), rapid response in severe hyperthyroidism, etc. It will be up to patients, physicians and insurance companies to set the value for such interventions.

Other challenges will differ according to the agent used:

- Therapeutics blocking the TSHR–TRAb interaction will likely block to some extent the TSHR–TSH interaction, leading to hypothyroidism. A ‘block and replace’ regimen may therefore be needed for these patients in order to ensure a stable euthyroid response. In addition, blocking the TSHR in extrathyroidal tissues (e.g., adipose tissue, bone gonads) may have unforeseen effects, as the role of the receptor outside the thyroid has not yet been clearly delineated. In addition, the interference of the therapeutic TSHR inhibitory antibodies with the measurements of the patient’s pathogenic TSHR antibodies will need to be further investigated. In the phase 1 study of the TSHR antagonist, TRAb titers increased rapidly with the administration of the higher doses of the K1-70 monoclonal antibody (57).
- For small molecule TSHR antagonists, the challenge relates to the similarity between the TSHR and LH/FSH receptors, and thus the need to ensure excellent specificity to the prior and avoid disruption of the sex hormone regulatory loop. Potential off-target effects on other yet unknown GPCRs with such agents are a possible risk, likely not shared with TSHR-blocking antibodies as evidenced by the limited data with K1-70 (55).
- The new agents that block FcRn introduce a different set of challenges. How fast thyroid function changes in response to TRAb clearance will determine the rapidity of methimazole taper and the frequency of thyroid function testing. It is possible that there is significant variation in rates of TRAb clearance/normalization in different individual patients, in which case it will be hard to develop uniform methimazole dosing and thyroid function testing protocols.
- Similar challenges will apply to tolerogenic therapy use, should it be further developed. In addition, for the latter, different apitopes may need to be developed for different MHC allelotypes, adding more complexity to the issues above.

### Management of GD in the presence of TED

If new agents that aim to decrease the production or increase the clearance of TSHR antibodies or block TSHR signaling become viable therapies, they will have the potential to simultaneously treat GD and TED. In that context, the collaboration between endocrinologists and ophthalmologists will be more important than ever. Joint thyroid eye clinics providing care for the broad TSHR autoimmunity spectrum will be the ideal setting for patient management; however, these are quite scarce outside of Europe. In the absence of these clinics, easy referral pathways, including virtual evaluations/discussions, and some level of cross-training will be necessary to ensure patient safety and optimal patient care. Endocrinologists treating GD will need to become more comfortable assessing and managing TED, particularly when it comes to systemic medical therapies, and similarly, ophthalmologists treating TED will need to familiarize themselves with the basics of thyroid dysfunction management. The new therapies will hopefully promote further multidisciplinary clinical care and more research collaborations between the two disciplines. Finally, in patients with GD that have co-existent TED, and vice versa, the choice of therapy will be influenced by efficacy against both diseases.

## Conclusion

The treatment of GD is seeing the shift from ablative therapy to ATDs, with data indicating this to be the dominant first therapy in most regions worldwide. The likelihood of remission can be more clearly defined with the use of GREAT and GREAT+ scores. If remission is not likely, that is no longer deterring patients and clinicians from continuing ATD therapy long term, as the benefit of preserving the thyroid and endogenous thyroid hormone production is becoming increasingly apparent.

We are witnessing, with high hope, the interest of the research community shifting toward manipulating the autoimmune response, either through inducing tolerance to the TSHR, eliminating the TRAb, or blocking the TSHR at the extracellular or the transmembrane domain. These approaches will hopefully allow a rapid resolution of clinical hyperthyroidism and its associated goiter, while also positively impacting TED and PTD, if present simultaneously. The added benefit in patients having isolated thyroid autoimmunity will be the expected decrease or elimination of the risk of TED, PTD and fetal/neonatal thyrotoxicosis. The new agents will nevertheless require adjustments to our disease monitoring practices and possibly utilization of a ‘block and replace’ principle with some of these therapies. In addition, we hope to see a more individualized approach to thyroid level testing and ATD dose adjustments through the utilization of technology and biomarkers already within the reach of today’s wearable devices.

## Declaration of interest

CD served as a consultant for Horizon/Amgen, Immunovant, Septerna, Argenx, and Third Rock Ventures. She participated in a research funded by Sling Therapeutics and Viridian Therapeutics. MNS’s institution received research grants from Tourmaline, Immunovant, and Lassen, and he received consulting fees from Third Rock Ventures, Avilar, Septerna, Minghui Pharma, Genentech, Tshaka Bio, Lassen and ArgenX.

## Funding

This work did not receive any specific grant from any funding agency in the public, commercial, or not-for-profit sector.

## Author contribution statement

Both authors have made substantial contributions to the design of the work, acquisition of data and drafting of the manuscript, and both gave final approval of the version to be published.
